# The study of non-Newtonian nanofluid with hall and ion slip effects on peristaltically induced motion in a non-uniform channel

**DOI:** 10.1039/c7ra13188g

**Published:** 2018-02-20

**Authors:** Sara I. Abdelsalam, M. M. Bhatti

**Affiliations:** Basic Science, Faculty of Engineering, The British University in Egypt Al-Shorouk City Cairo 11837 Egypt sara.abdelsalam@bue.edu.eg siabdelsalam@caltech.edu siabdelsalam@yahoo.com; Division of Chemistry and Chemical Engineering, California Institute of Technology Pasadena CA 91125 USA; Shanghai Institute of Applied Mathematics and Mechanics, Shanghai University Shanghai 200072 China

## Abstract

In this study, we considered the unsteady peristaltic motion of a non-Newtonian nanofluid under the influence of a magnetic field and Hall currents. The simultaneous effects of ion slip and chemical reaction were also taken into consideration. The flow problem was suggested on the basis of the continuity, thermal energy, linear momentum, and nanoparticle concentration, which were further reduced with the help of Ohm's law. Mathematical modelling was executed using the lubrication approach. The resulting highly nonlinear partial differential equations were solved semi-analytically using the homotopy perturbation technique. The impacts of all the pertinent parameters were investigated mathematically and graphically. Numerical calculations have been used to calculate the expressions for the pressure increase and friction forces along the whole length of the channel. The results depict that for a relatively large value of the Brownian parameter, the chemical reaction has a dual behaviour on the concentration profile. Moreover, there is a critical point of the magnetic parameter at which the behaviours of the pressure increase and friction forces are reversed for progressive values of the power law index. The present investigation provides a theoretical model that estimates the impact of a wide range of parameters on the characteristics of blood-like fluid flows.

## Abstract 2

In this study, we considered the unsteady peristaltic motion of a non-Newtonian nanofluid under the influence of a magnetic field and Hall currents.

## Graphical abstract

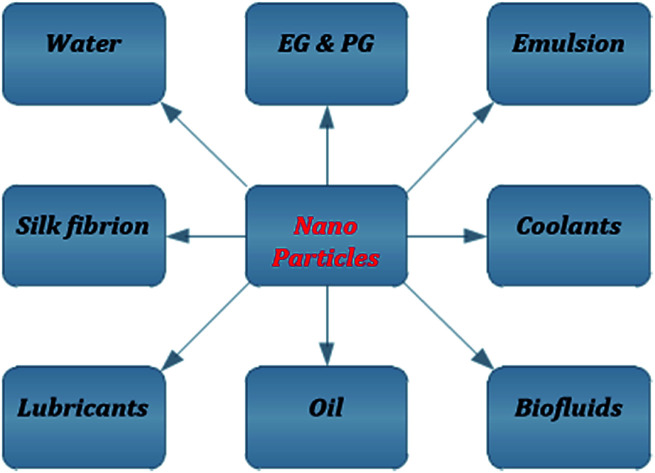

## 1. Introduction

During the recent years, the study of peristaltic flow has become an increasing interest for various researchers due to its efficient phenomena for the transport of fluids in different biological systems. It is a phenomenon in which a sinusoidal wave arises due to the proportional shrinkage and relaxation of smooth muscles in a human body. In particular, the peristaltic flow is involved in the transport of urine through the kidney to the bladder, transport of cilia, locomotion of spermatozoa (in the male reproductive tract), motion of chyme (in the gastrointestinal tract), motion of ova (in the fallopian tubes), and in the vasomotion of tiny blood vessels. In industry, peristaltic mechanism is very beneficial in transporting different biological fluids such as sanitary and corrosive fluids. For this purpose, many devices, such as heat lung machines, roller pumps, cell separators, and finger pumps, have been introduced in biomedical engineering that follow the fundamentals of peristaltic mechanism. Due to the numerous applications of the peristaltic flow, several researchers investigated the mechanism of peristalsis in different media. For instance, Mekheimer^1^ studied the motion of couple stress fluid due to the peristaltic waves through a non-uniform channel. Later, Mekheimer^2^ extended the previous problem influenced by the magnetic field considering blood as a couple stress fluid and obtained the exact solutions. The nonlinear peristaltic flow under the influence of a magnetic field through a uniform planar conduit was discussed by Hayat *et al.*^3^ Ellahi *et al.*^4^ investigated the peristalsis of three-dimensional motion of a non-Newtonian fluid in a rectangular canal. The peristaltic flow of the non-Newtonian Williamson fluid with compliant walls was investigated by Ellahi *et al.*^5^ He further analysed the impacts of the wall tension and damping and obtained the series solution with the help of the perturbation technique. Nadeem *et al.*^6^ studied the three-dimensional peristaltic motion of a Jeffrey fluid in a duct having flexible walls and obtained the exact solutions. Mekheimer *et al.*^7^ studied the influence of the relaxation time of a Maxwell fluid together with the MHD peristaltic transport in a microchannel. Some further similar investigations on this topic can be found in the literature.^8–10^

**Fig. 1:**
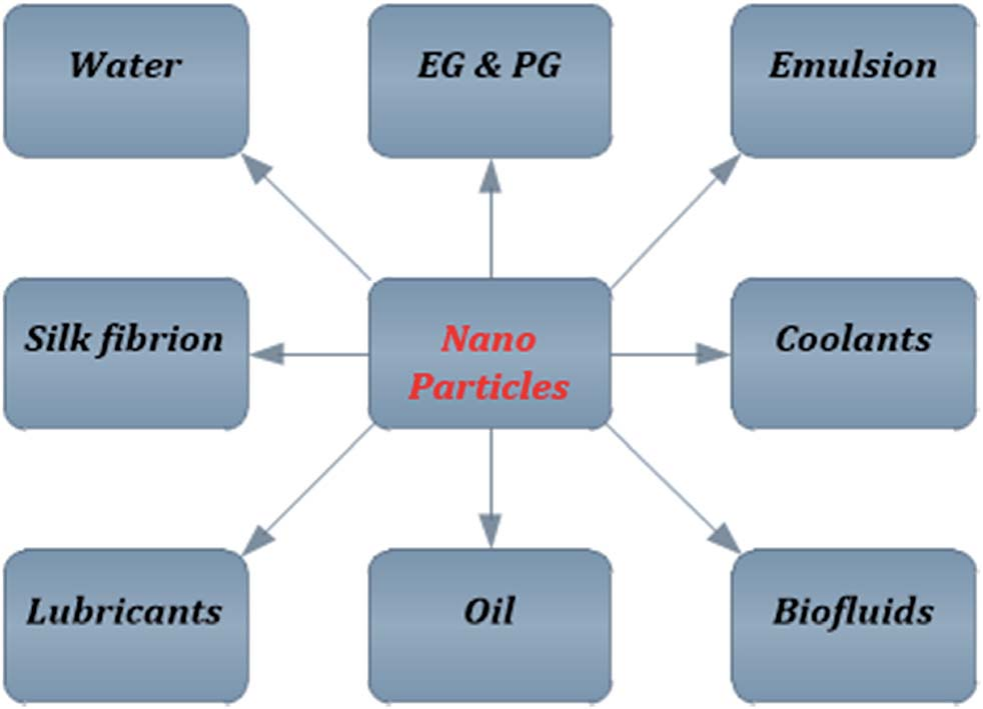
Combination of NPs with different base fluids.

In fluid dynamics, a new branch has been introduced namely nanofluid dynamics, which has many applications in biology, medical science, energetics, and engineering processes. Nanotechnology introduces the creation and usage of numerous substances having the nanoscale dimensions from 1 to 100 nm. Basically, a nanofluid (NF) is a fluid that is amalgamated by scattering the nanoparticle (NP) in the base fluid such as body fluids, natural/artificial lubes, and water. Choi^11^ was the first who initially concluded that the impact of the nanofluid phenomena was to enhance the energy performance. Although the basic concept of NFs was introduced in the 19^th^ century by a well-known scientist James Clark (a Scottish theoretical physician), later the term nanofluid was introduced officially by Choi. Lee *et al.*^12^ investigated the room-temperature thermal conductivity of NFs as a new class of fluids that was organized by dispersing NPs in water and ethylene glycol (EG). The non-Newtonian NF was investigated by Ellahi *et al.*^13^ with Reynolds' model and Vogel's model using the homotopy method. Nanofluid particles are made up of metals, oxides, nitrides, or carbides having very small diameters (<100 nm). The base fluids can be the EG, lubricants, propylene glycol (PG), biofluids, coolants, emulsion, water, or silk fibroin. Fig. 1 shows the combination of different NPs with base fluids. Nanofluids are applicable and helpful in the understanding of various phenomena such as the enhancement/minimization of the magnitude of heat transfer systems, minimal clogging, miniaturization of the systems, and microchannel clogging.

Various researchers studied the combined mechanism of peristalsis with NF through different geometrical aspects. For instance, Akbar *et al.*^14^ studied the peristaltic NF flow in an irregular tube. Akbar *et al.*^15^ examined numerically the peristalsis of Williamson fluid in an asymmetric channel. Nadeem *et al.*^16^ discussed the NF peristalsis in an eccentric conduit with heat and mass transfer. Ellahi *et al.*^17^ studied theoretically the peristaltic mechanism of Prandtl nanofluid through a rectangular duct. Nadeem *et al.*^18^ presented a mathematical formulation for the peristaltic motion of a non-Newtonian fluid with NPs. The peristaltic flow of NF having carbon NPs through a permeable channel under the influence of an induced magnetic field was investigated by Akbar *et al.*^19^ Few more relevant studies can be found in the literature.^20–22^

The existing fashion in the applications of MHD is towards the strong magnetic fields to take the effect of electromagnetic force into consideration. Consequently, the Hall and ion slip effects are crucial since they have exceptional influence on the current density. The Hall effect and ion slip effect have numerous applications, especially if incorporated with heat transfer, such as in Hall accelerators, refrigeration coils, heating elements, MHD accelerators, and power generators. Moreover, the study of the influence of the magnetic field along with the Hall and ion slip effects on the blood flow in an artery has been found to be very helpful and applicable in magnetic resonance angiography (MRA). This helps to create the images of arteries to explore the existence of stenosis or any other conditions in the arteries of the brain, abdomen, thorax, and kidneys. Magnetic resonance imaging (MRI) is also involved in other applications that involve pumping of blood, hyperthermia, cancer therapy, and magnetic drug targeting. Since the implementation of these applications provides exclusive capabilities to improve the mechanism of peristalsis uses, researchers have devoted much effort towards studying the peristaltic nanofluid with the magnetic field in different conduits. For instance, El Koumy *et al.*^23^ studied the peristaltic motion of a Maxwell fluid under the influence of a strong magnetic field and accordingly the Hall effect through a conduit. Asghar *et al.*^24^ investigated the simultaneous effects of Hall and ion slip along with the ohmic and viscous heating on the peristaltic motion through different ducts. Hayat *et al.*^25^ considered the Hall and ion slip effects on the peristaltic phenomenon of a non-Newtonian Carreau–Yasuda fluid model. Abbasi *et al.*^26^ considered the peristaltic motion of a silver–water nanofluid with the Hall and ion slip effects. They, in addition, considered the ohmic heating and wall characteristics such as the tension of elasticity and damping phenomenon. More comprehensive treatments about magnetic field models can be found in the outlined ref. 27–33.

Considering the abovementioned discussion, the primary motivation of the present study was to extend our interest in studying the peristaltic motion of a hyperbolic tangent fluid with the effects of Hall and ion slip through a non-uniform channel taking the chemical reaction into consideration. To the best of our knowledge, this model has not been investigated in any of the referenced state-of-the-art reviews before. The system of equations describing the problem is formed by following the approach of the long wavelength and creeping flow regime. The resulting governing nonlinear partial differential equations have been solved by means of the HPM (homotopy perturbation method). The impacts of all the emerging parameters have been discussed in details with the help of the graphs.

## 2. Mathematical formulation

**Fig. 2:**
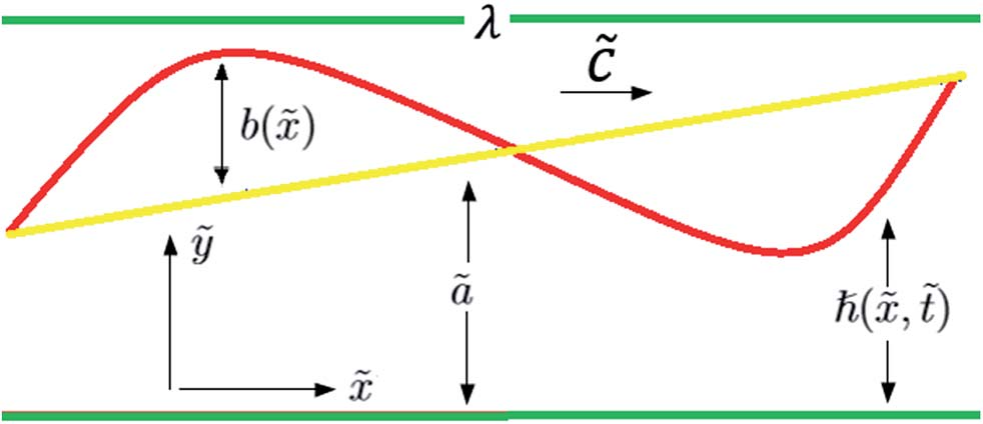
Schematic of the flow problem.

We considered the peristaltic flow of a blood-like incompressible, hyperbolic tangent, and electrically conducting NF under the effect of an externally applied magnetic field. A flow through the 2D non-uniform channel is induced due to the propagation of sinusoidal waves along its walls. The hydromagnetic flow of the nanofluid is considered unsteady and irrotational. We choose the Cartesian coordinate system in a way that *x̃*-axis is taken along the channel length and *ỹ*-axis is normal to it, as shown in Fig. 2. The geometry of the peristaltic walls can be described by1
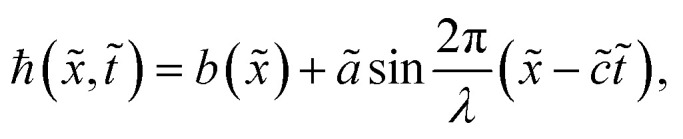
where*b*(*x̃*) = *b*_0_ + *K̄x̃*,

The generalized form of Ohm's law taking the Hall and ion slip effects into consideration can be written as2
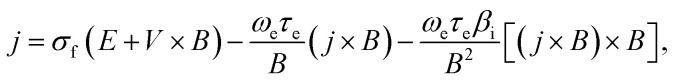


Solving eqn (2), we obtain3
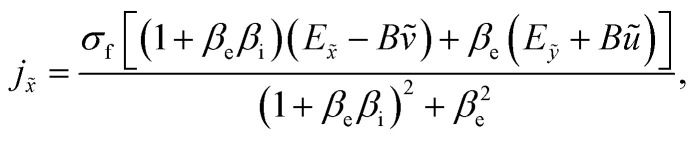
and4
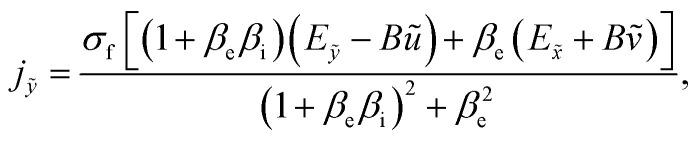
where *β*_e_ = *ω*_e_*τ*_e_.

The equations of motion governing the flow along with the thermal energy, continuity, and nanoparticle fraction for the blood NF can be written as^34^5
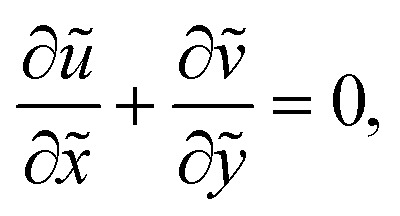
6
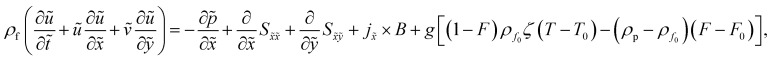
7
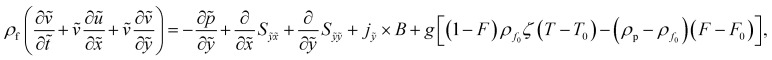
8
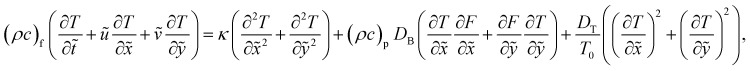
9
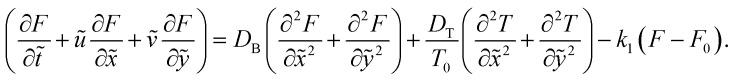


The stress tensor for hyperbolic tangent fluid is defined as10
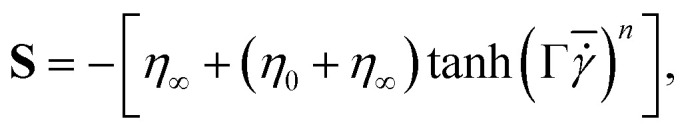


In the abovementioned equation, we have considered *η*_∞_ = 0 and 
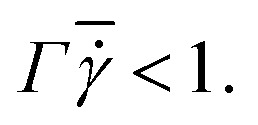
 Accordingly, the stress tensor can be rewritten as11
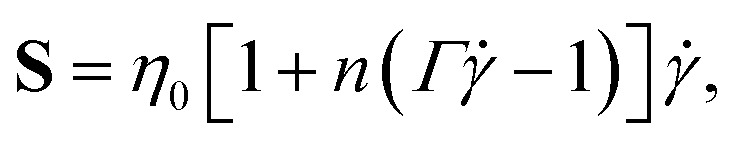


Defining the dimensionless quantities as
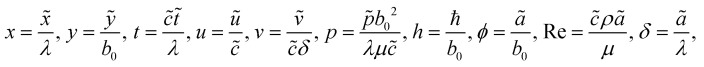

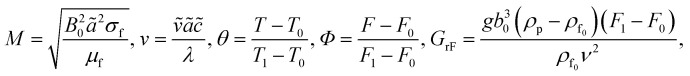
12
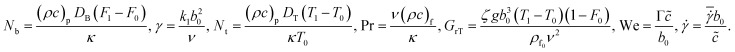


We followed the creeping flow proposition such that the half-width of the conduit was taken small as compared to the peristaltic wavelength. We further speculated that the Reynolds number is low. These assumptions are extensively used in many peristalsis analyses.^30,32,35–38^ These approximations are considered in many biological tracts such as in the transport of enzymes to the duodenum. Using eqn (12) in eqn (3)–(10), we obtained the reduced system of equations in the following form:13
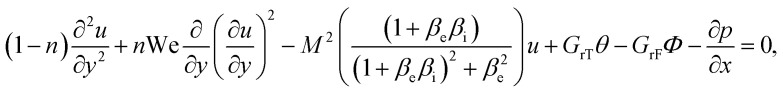
14
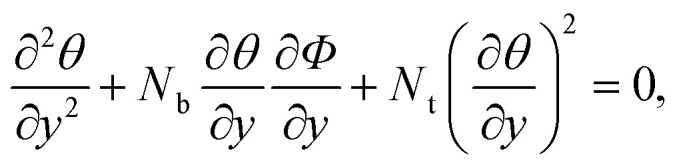
15
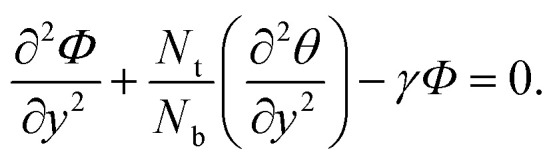
with corresponding boundary conditions as16
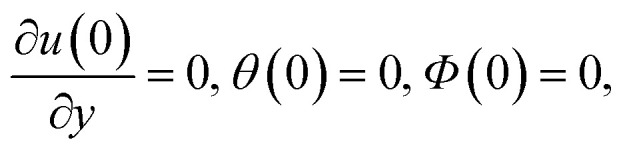

17 *u*(*h*) = 0, *θ*(*h*) = 1, *Φ*(*h*) = 1,

where 
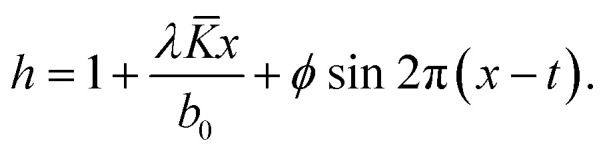


## 3. Method of solution

In this section, we attempted to solve the aforementioned non-linear couple of partial differential equations by means of the HPM. The homotopy for eqn (13)–(15) can be written as18
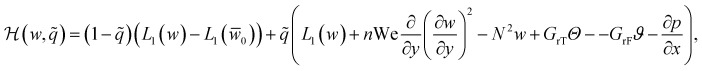
19
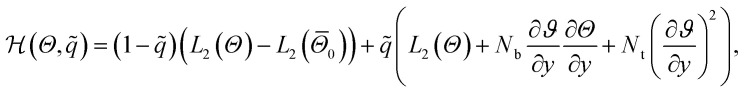
20
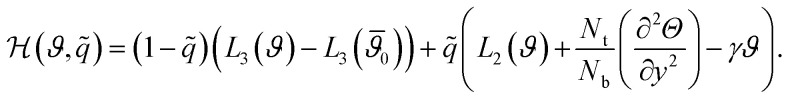


The linear operators *L*_1_, *L*_2_, and *L*_3_ are suggested in the next forms21
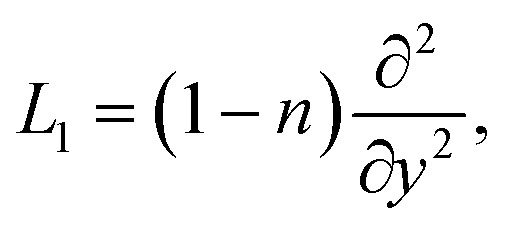
22
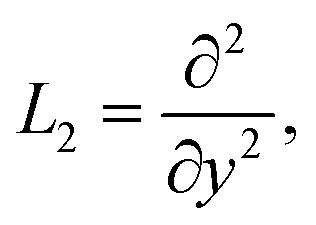
23
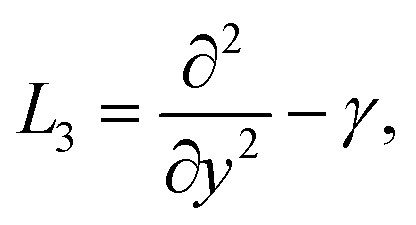


Moreover, we defined the initial guess for the abovementioned linear operators as24
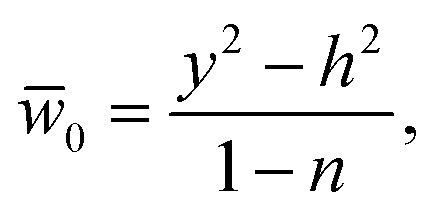
25
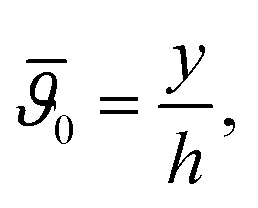
26
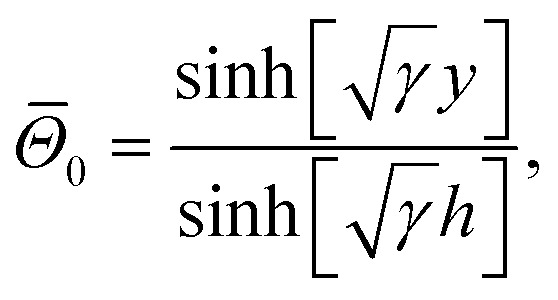


Defining the following expansions

27 *w*(*x*,*y*) = *w*_0_(*x*,*y*) + *q̃w*_1_(*x*,*y*) + *q̃*^2^*w*_2_(*x*,*y*) + …,

28 *Θ*(*x*,*y*) = *Θ*_0_(*x*,*y*) + *q̃Θ*_1_(*x*,*y*) + *q̃*^2^*Θ*_2_(*x*,*y*) + …,

29 *ϑ*(*x*,*y*) = *ϑ*_0_(*x*,*y*) + *q̃ϑ*_1_(*x*,*y*) + *q̃*^2^*ϑ*_2_(*x*,*y*) + …,

By substituting eqn (27)−(29) into eqn (18)−(20) and matching the like powers of *q̃*, a linear system of differential equations, along with their corresponding boundary conditions, was obtained. With reference to the scheme of HPM, we deduced the solution as *q̃* → 1, and we obtained

30 *u*(*x*,*y*) = *w*(*x*,*y*) = *w*_0_(*x*,*y*) + *w*_1_(*x*,*y*) + *w*_2_(*x*,*y*) + …,

31 *θ*(*x*,*y*) = *Θ*(*x*,*y*) = *Θ*_0_(*x*,*y*) + *Θ*_1_(*x*,*y*) + *Θ*_2_(*x*,*y*) + …,

32 *Φ*(x,y) = *ϑ*(*x*,*y*) = *ϑ*_0_(*x*,*y*) + *ϑ*_1_(*x*,*y*) + *ϑ*_2_(*x*,*y*) + …,

The solutions of the temperature profile, nanoparticle concentration, and velocity profile can simply be written as33
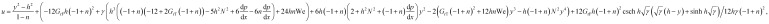
34
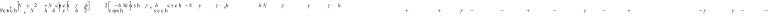
35
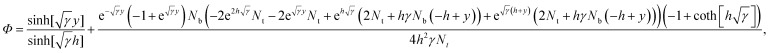
where. 
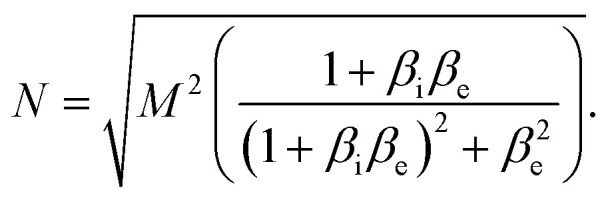


The instantaneous volume flow rate can be determined through the expression36
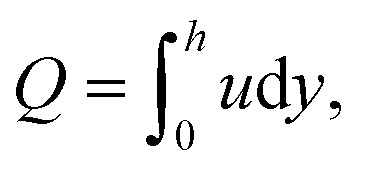


Thus, we can obtain the expression of the pressure gradient, d*p*/d*x*, after solving the latter equation. Hence, the dimensionless forms of pressure increase, Δ*p*_*L*_, and friction force, Δ*f*_*L*_, by the wall are given by37
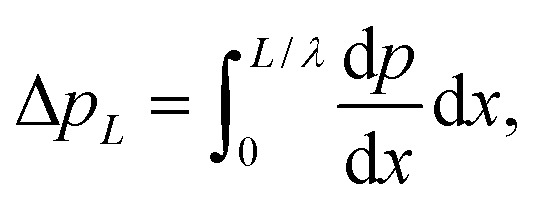
38
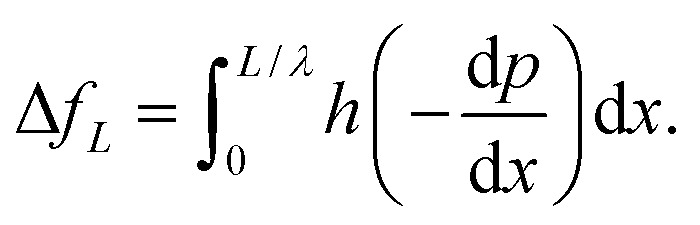
where *L* is the non-uniform channel length.

## 4. Numerical results and discussion

In this part, we have discussed the theoretical significance of the developed physical expressions that are involved in the problem based on the current study. A Mathematica toolbox has been used to explore the outcomes arising due to the existence of the Brownian parameter *N*_b_, thermophoresis parameter *N*_*t*_, chemical reaction parameter *γ*, ion slip parameter *β*_i_, Hall parameter *β*_e_, basic density Grashof number *G*_rF_, thermal Grashof number *G*_rT_, power law index *n*, magnetic parameter *M*, Weissenberg number We, and the average time flow *Q̄* into the flow field. More specifically, we investigated their influence on the distributions of temperature *θ*, concentration *Φ*, and velocity *u*, as well as the pressure increase Δ*p*_*L*_ and friction force Δ*f*_*L*_. In the subsequent figures, the red, blue, and green coloured curves represent the variations of the given variable with the indicated parameter in an ascending order. Same is applicable to the variations of the solid and dashed lines where the solid line indicates a smaller value of the parameter under consideration. We consider that *Q*(*x*,*t*) is the instantaneous volume flow rate, which is cyclic having (*x* − *t*) cycle and hence can be written as*Q*(*x*,*t*) = *Q̄* + ϕ sin 2π(*x* − *t*).where *Q̄* expresses the average of the time flow over one wave cycle.

**Fig. 3:**
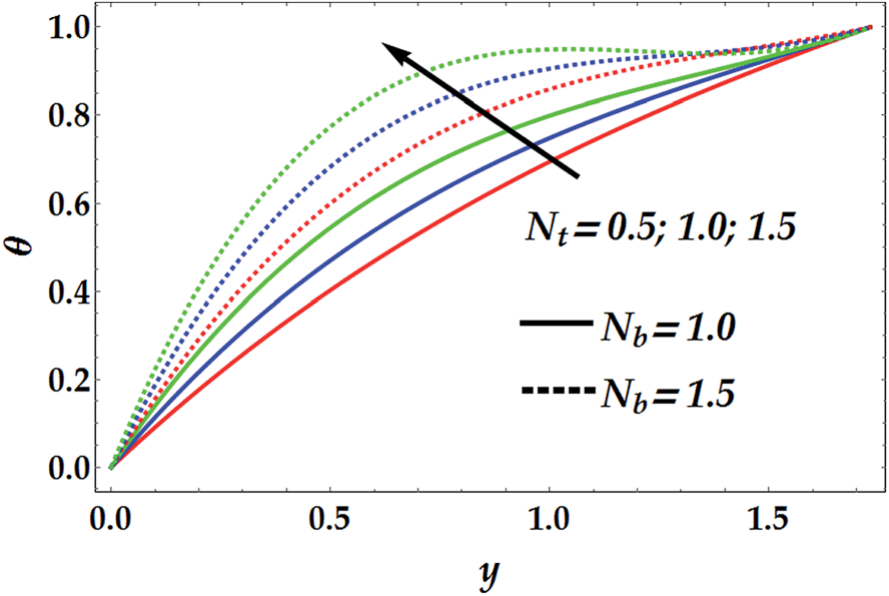
Temperature distribution for various values of *N*_b_ and *N*_t_ at We = 0.01, *M* = 1, *G*_rT_ = 0.5, *G*_rF_ = 0.6, *γ* = 0.1, and *n* = 2.

**Fig. 4:**
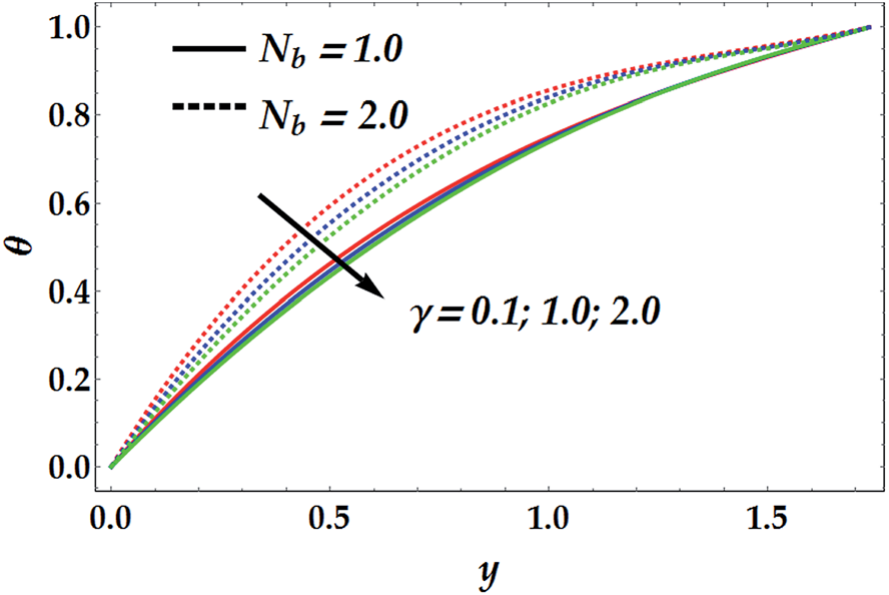
Temperature distribution for various values of *N*_b_ and *γ* at *N*_t_ = 0.5, We = 0.01, *M* = 1, *G*_rT_ = 0.5, *G*_rF_ = 0.6, and *n* = 2.

**Fig. 5:**
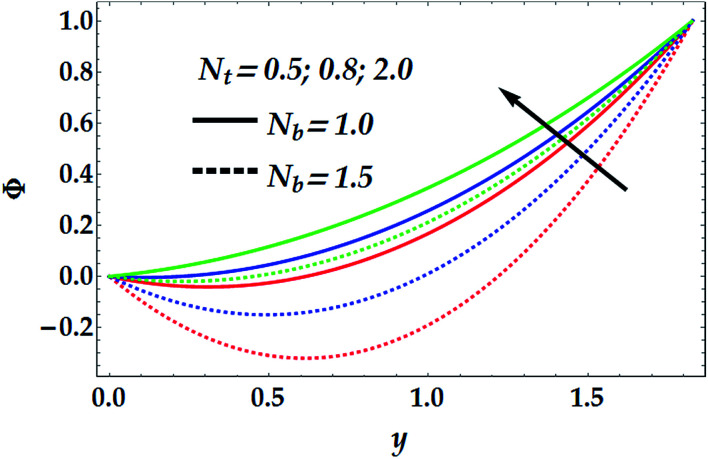
Concentration distribution for various values of *N*_t_ and *N*_b_ at We = 0.01, *M* = 1, *G*_rT_ = 0.5, *G*_rF_ = 0.6, *γ* = 0.1, and *n* = 2.

**Fig. 6:**
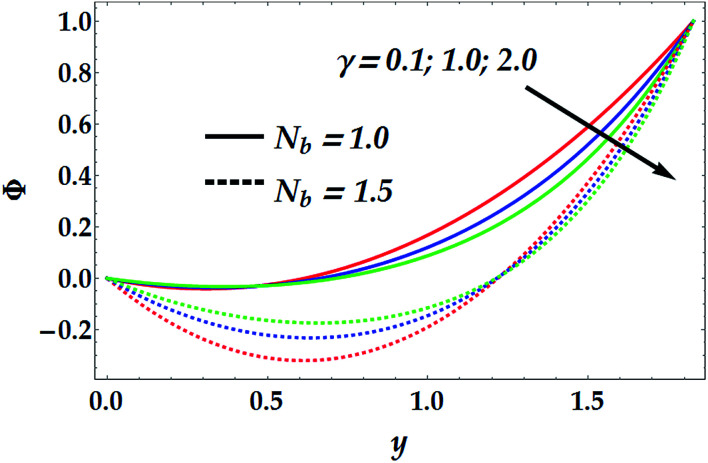
Concentration distribution for various values of *N*_b_ and *γ* at *N*_t_ = 0.5, We = 0.01, *M* = 1, *G*_rT_ = 0.5, *G*_rF_ = 0.6, and *n* = 2.

Fig. 3−6 provide insight into the changes in the behaviour of the temperature and concentration distributions on the nanofluid that occur due to changes in the values of the chemical reaction parameter *γ*, the thermophoresis parameter *N*_t_, and the Brownian parameter *N*_b_. Fig. 3 examines the dependence of *θ* that is plotted with y for different values of *N*_b_ and *N*_t_. The figure shows that the temperature profile *θ* is semi-parabolic, and there is a considerable increase in *θ* upon increasing both *N*_b_ and *N*_t_. The possible reason is that the Brownian motion causes the nanoparticles to rearrange, forming a blend, which increases the thermal conductivity. Further, eqn (14) and (15) show that the temperature profile is commensurate with the thermophoresis parameter. The latter result may be important in the event that a treatment requires an increase in the temperature of the tissues such as in the case of magnetic hyperthermia treatment where the major aim of hyperthermia is to increase the temperature of malignant tissues above 42 °C. On the other hand, it is shown in Fig. 4 that *θ* is reduced with an increase in *γ* for distinct values of *N*_b_. Further, it is shown that as *N*_b_ increases, the impact of *γ* becomes more significant. In this case, it must be taken into account that an increase in the Brownian parameter may result in an increase in the chemical reaction of the nanofluid temperature profile.

Fig. 5 depicts the influence of *N*_b_ and *N*_t_ on the concentration of nanofluid, *Φ*. It is noticed that *N*_b_ has a decreasing effect on *Φ* for different values for *N*_t_, whereas *N*_t_ shows a quite opposite effect on *Φ* for various values of *N*_b_. It is further shown that the Brownian parameter increases the impact of the thermophoresis in the nanofluid concentration profile. Fig. 6 illustrates the behaviour of *Φ* with various values of the chemical reaction *γ* at different values of the Brownian parameter. In this figure, it is shown that for a small value of *N*_b_ (=1), the chemical reaction seems to weakly affect *Φ* in the narrow part of the channel where *y* ∈ [0, 0.48], whereas *γ* tends to reduce the concentration profile afterwards. Conversely, for a larger value of *N*_b_ (=1.5), there is an obvious dual behaviour of *γ* on the concentration profile. That is, the concentration is enhanced in the region of *y* > 1.3, where *γ* enhances the fluid density, whereas it is reduced in the region of *y* < 1.3 due to the reduction in viscosity.

**Fig. 7:**
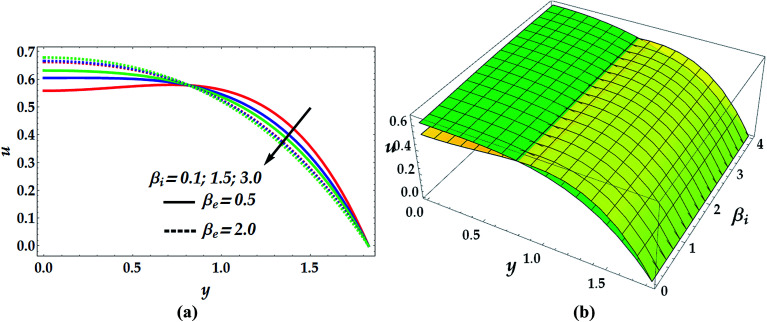
2D (a) and 3D (b) velocity distribution as a function of *y* for various values of *β*_i_ and *β*_e_ at We = 0.01, *M* = 1, *G*_rT_ = 0.5, *G*_rF_ = 0.6, *γ* = 0.1, and *n* = 2. For the 3D velocity profile: (i) yellow shading: *β*_e_ = 0.5, (ii) green shading: *β*_e_ = 2.

**Fig. 8:**
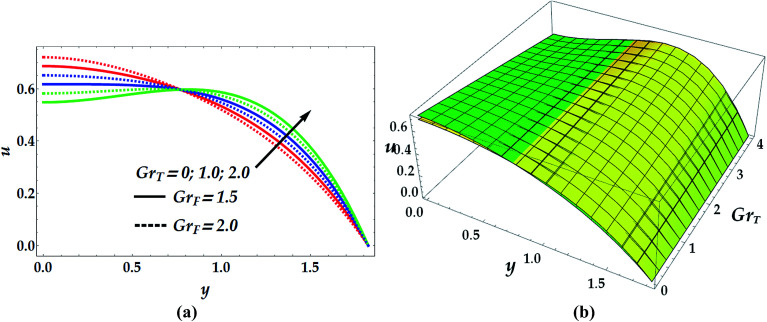
2D (a) and 3D (b) velocity distribution as a function of *y* for various values of *G*_rT_ and *G*_rF_ at *β*_e_ = 0.5, *β*_i_ = 0.6, We = 0.01, *M* = 1, *γ* = 0.1, and *n* = 2. For the 3D velocity profile: (i) yellow shading: *G*_rF_ = 1.5, (ii) green shading: *G*_rF_ = 2.

**Fig. 9:**
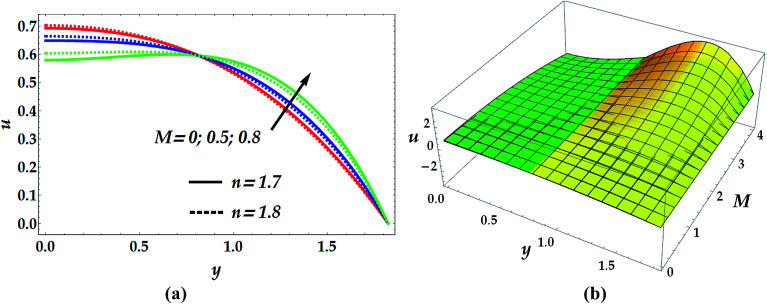
2D (a) and 3D (b) velocity distribution as a function of *y* for various values of *M* and *n* at *β*_e_ = 0.5, *β*_i_ = 0.6, We = 0.01, *G*_rT_ = 0.5, *G*_rF_ = 0.6, and *γ* = 0.1. For the 3D velocity profile: (i) yellow shading: *n* = 1.7, (ii) green shading: *n* = 1.8.

**Fig. 10:**
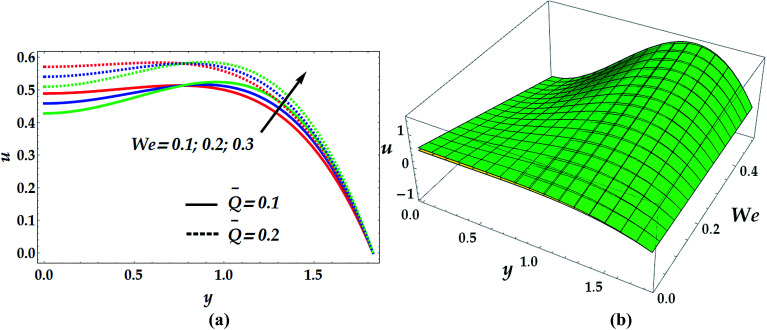
2D (a) and 3D (b) velocity distribution as a function of *y* for various values of We and *Q̅* at *β*_e_ = 0.5, *β*_i_ = 0.6, We = 0.01, *G*_rT_ = 0.5, *G*_rF_ = 0.6, *M* = 1, *n* = 2, and *γ* = 0.1. For the 3D velocity profile: (i) yellow shading (below): *Q̅* = 0.1, (ii) green shading *Q̅* = 0.2.

The two-dimensional and three-dimensional behaviour of the velocity profile are displayed in Fig. 7−10 for distinct values of the emerged parameters. Fig. 7 helps to elaborate the effects of the slip parameter, *β*_i_, and the Hall parameter, *β*_e_, on the velocity distribution. This figure reveals that the flow is accelerated for progressive values of *β*_i_ till a certain turning point at *y* = 0.8 of negligible slip effect from which the flow decreases substantially afterwards. Similarly, the impact of the Hall parameter is seen to accelerate the flow till the same point before it begins to lag. Fig. 8 demonstrates the influence of the basic density Grashof number *G*_rF_ and thermal Grashof number *G*_rT_ on the velocity profile. Physical interpretation of the behaviour of *G*_rF_ suggests that beyond a certain critical point (*y* = 0.78), the flow of the nanofluid decelerates with an increase in *G*_rF_. This has been predictable since a reduction in the Grashof number in the narrow part of the channel implies an increase in the viscosity causing deceleration in the velocity profile and *vice versa*. Conversely, the influence of *G*_rT_ is observed to reduce the velocity distribution till *y* = 0.78 from which the velocity increases markedly afterwards.

Fig. 9 illustrates the impact of the power law index *n* and the magnetic parameter *M* on the velocity profile. The examination shows that the flow of nanofluid decelerates when the value of *M* increases in the narrow part of the channel where *y* ∈ [0, 0.82]. The effect is quite opposite afterwards where the fluid flow is seen to be substantially increasing with an increase of *M*. It is also noticed that in the narrow part of the channel, the magnitude of velocity is higher in the absence of the magnetic parameter. Thus, the nanofluid velocity can be reduced in this part by the application of a strong magnetic field on the flow. Contrariwise, the influence of *n* on the velocity distribution is seen to be increasing in the narrow part and decreasing afterwards. Fig. 10 helps to demonstrate the influence of We and *Q̄* on the velocity profile. The inspection of this graph reveals that the flow decelerates for progressive values of We in the narrow part of the channel until *y* = 0.82, where its effect on *u* is negligible. Later on, the behaviour of the flow is reversed with an increase in We. However, the velocity profile is seen to be remarkably accelerated with an increment in *Q̄*.

**Fig. 11:**
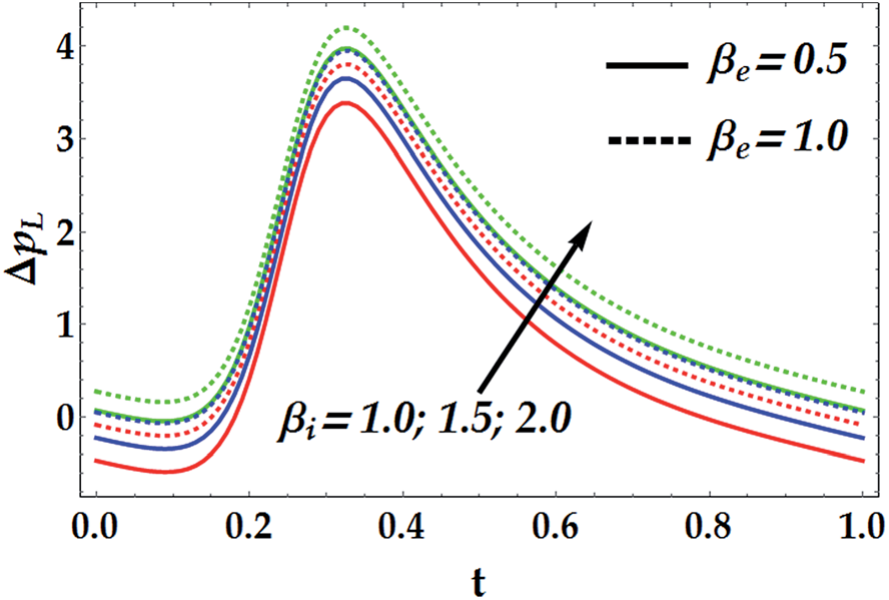
Pressure increase for various values of *β*_e_ and *β*_i_ at We = 0.01, *M* = 1, *γ* = 0.1, *G*_rT_ = 0.5, *G*_rF_ = 0.6, and *n* = 2.

**Fig. 12:**
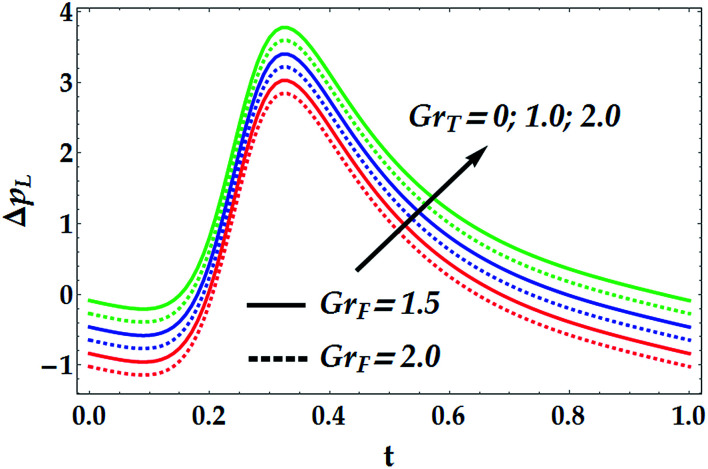
Pressure increase for various values of *G*_rF_ and *G*_rT_ at We = 0.01, *M* = 1, *γ* = 0.1, *β*_i_ = 0.6, *β*_e_ = 0.5, and *n* = 2.

**Fig. 13:**
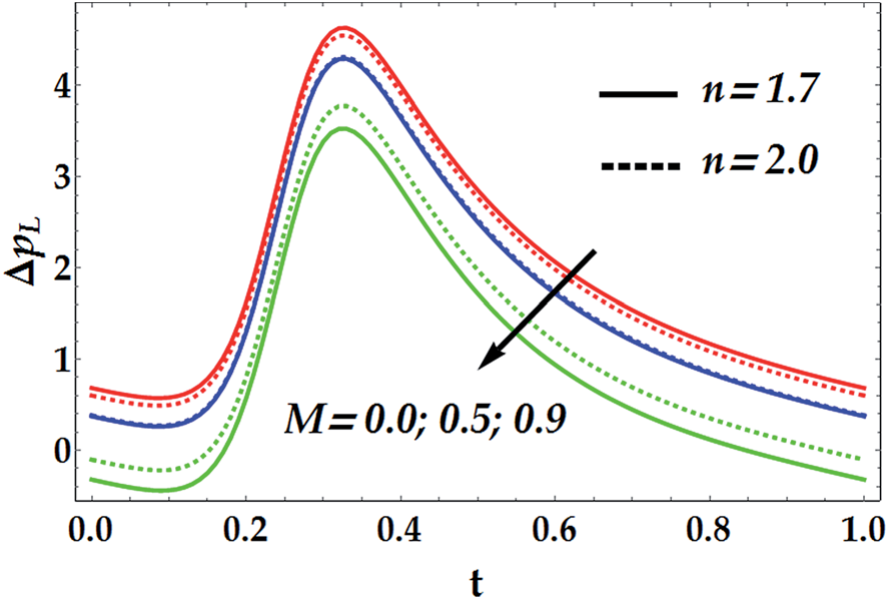
Pressure increase for various values of *n* and *M* at We = 0.01, *γ* = 0.1, *G*_rT_ = 0.5, *G*_rF_ = 0.6, *β*_i_ = 0.6, *β*_e_ = 0.5, and *n* = 2.

**Fig. 14:**
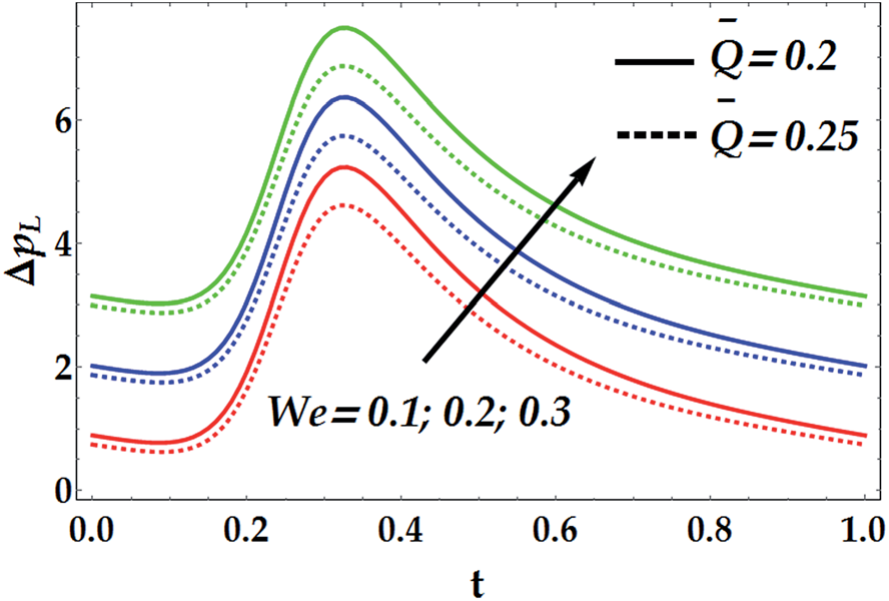
Pressure increase for various values of We and *Q̅* at We = 0.01, *γ* = 0.1, *G*_rT_ = 0.5, *G*_rF_ = 0.6, *β*_i_ = 0.6, *β*_e_ = 0.5, *M* = 1, and *n* = 2.

To study the influence of the pertinent parameters on the pressure increase Δ*p*_*L*_, Fig. 11−14 have been plotted. Fig. 11 demonstrates the effect of the slip parameter *β*_i_ and the Hall parameter *β*_e_ on the pressure increase. It is deduced from this graph that Δ*p*_*L*_ is reinforced with escalating both *β*_i_ and *β*_e_. It is further noticed that Δ*p*_*L*_ is almost unperturbed by the variations in *β*_i_ and *β*_e_ in the interval *t* ∈ [0.22, 0.3], whereas the pressure increase attains its maximum value subsequently at *t* = 0.34. The fact that pressure increase is small in some intervals can be interpreted as the flow can facilely pass without imposition of large pressure, whereas to retain the same flux, large pressure is required. Fig. 12 and 13 are shown to investigate the effects of *G*_rF_, *G*_rT_, *M*, and *n* on the pressure increase Δ*p*_*L*_. A close look to the graphs reveals that Δ*p*_*L*_ increases with the progressive values of *G*_rT_, whereas it decreases with an increase in *G*_rF_ and *M* with a maximum value occurring at *t* = 0.32. It is also shown that *M* = 0 causes *n* to have a decreasing effect on the pressure increase till it reaches a critical value of *M* (= 0.5) where *n* weakly affects Δ*p*_*L*_. Afterwards, the behaviour of Δ*p*_*L*_ is totally reversed to be increasing with *n* at a higher value of *M*. It is recognized that the pressure increase is higher in the absence of *M*. This phenomenon symbolizes the fact that the pressure can be controlled by suitably applying the magnetic field on the flow. This is an important factor in the use of magnetic field in physiology as any abrupt change in the intensity of the applied magnetic field can cause severe changes in the systolic/diastolic readings of the patient exposed to the magnetic field. Fig. 14 shows the influence of We and *Q̄* on Δ*p*_*L*_. Evidently, We has an increasing effect on Δ*p*_*L*_, whereas *Q̄* has a decreasing effect on it. Further, the pressure increase attains its maximum value at *t* = 0.35.

**Fig. 15:**
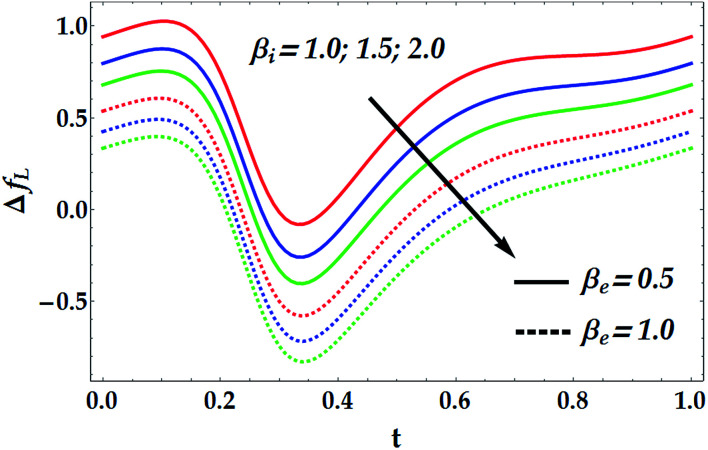
Friction forces for various values of *β*_e_ and *β*_i_ at We = 0.01, *M* = 1, *γ* = 0.1, *G*_rT_ = 0.5, *G*_rF_ = 0.6, and *n* = 2.

**Fig. 16:**
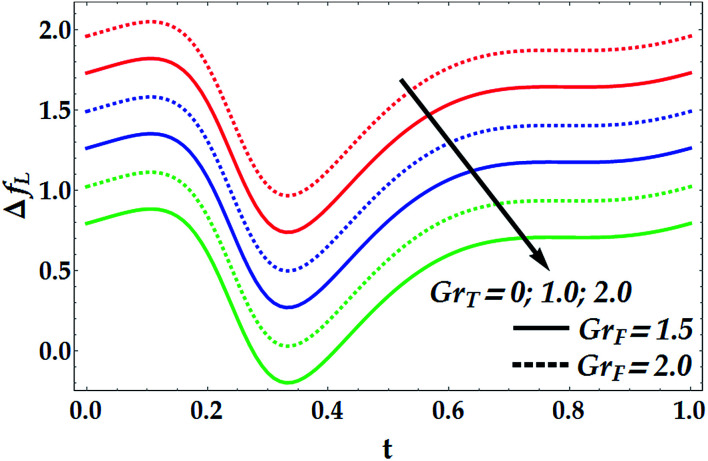
Friction forces for various values of *G*_rT_ and *G*_rF_ at We = 0.01, *M* = 1, *γ* = 0.1, *β*_i_ = 0.6, *β*_e_ = 0.5, and *n* = 2.

**Fig. 17:**
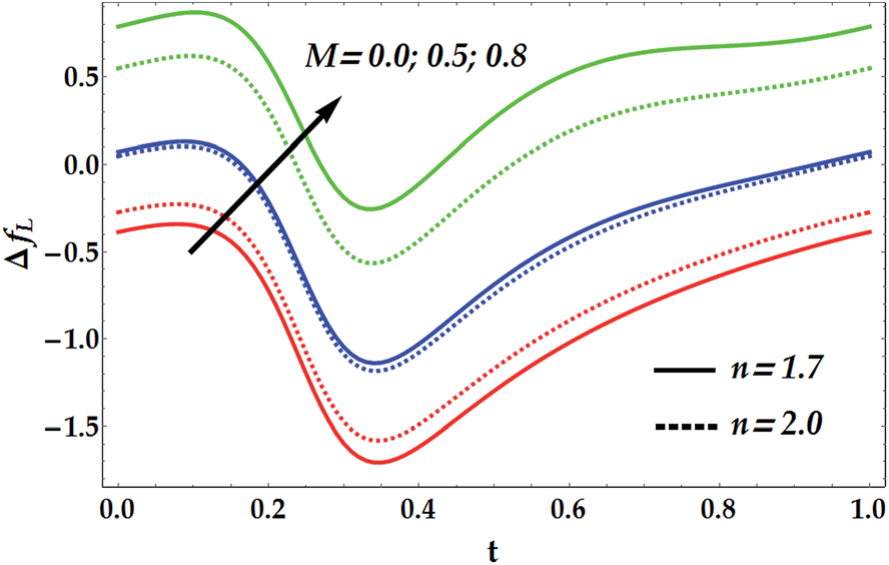
Friction forces for various values of *M* and *n* We = 0.01, *γ* = 0.1, *G*_rT_ = 0.5, *G*_rF_ = 0.6, *β*_i_ = 0.6, and *β*_e_ = 0.5.

**Fig. 18:**
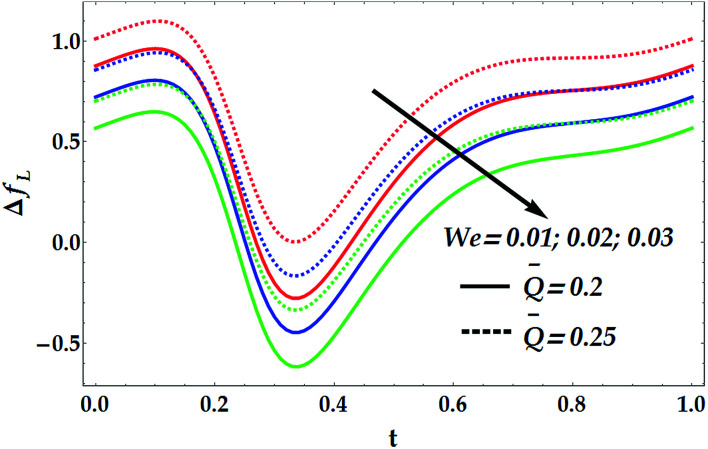
Friction forces for various values of We and *Q̅* at We = 0.01, *γ* = 0.1, *G*_rT_ = 0.5, *G*_rF_ = 0.6, *β*_i_ = 0.6, and *β*_e_ = 0.5.

Fig. 15−18 provide a perspective of the influence of the parameters under consideration on the friction force Δ*f*_*L*_. Fig. 15 provides insight into the effect of the slip parameter *β*_i_ and the Hall parameter *β*_e_ on Δ*f*_*L*_. It is seen from this figure that Δ*f*_*L*_ decays prominently with the progressive values of *β*_i_ and *β*_e_. It is further observed that Δ*f*_*L*_ is minimum at *t* = 0.34. Different variations are observed for the effects of the basic Grashof number *G*_rF_ and thermal Grashof number *G*_rT_ on Δ*f*_*L*_ throughout the domain *t* ∈ [0, 1], as seen in Fig. 16. It is observed that *G*_rF_ has an increasing effect on the friction force, whereas *G*_rT_ has a decreasing effect on it. It is also noticed that Δ*f*_*L*_ attains its minimum at *t* = 0.33. Fig. 17 helps to demonstrate the influence of the Hartmann number and power law index on Δ*f*_*L*_. It is concluded that Δ*f*_*L*_ increases with an increase in *M*. Moreover, it is shown that *M* = 0 causes *n* to have a decreasing effect on the friction force till it reaches a critical value of *M* (= 0.5) where *n* weakly affects Δ*f*_*L*_. Afterwards, the behaviour of Δ*f*_*L*_ is totally reversed to be increasing with *n* at a higher value of *M*. In addition, it is seen that the friction force is lower in the absence of *M*, and the minimum value of Δ*f*_*L*_ takes place at *t* = 0.33. Fig. 18 depicts the variations in Δ*f*_*L*_ due to the changes in We and *Q̄*. Obviously, the friction force is seen to be enhanced noticeably for the higher values of *Q̄*, whereas it decays with an increase in We. It is also shown that Δ*f*_*L*_ attains its minimum at *t* = 0.33.

## 5. Conclusions

In this study, the peristaltic flow of a blood-like non-Newtonian (hyperbolic tangent) NF is investigated in a non-uniform channel to study the mathematical results under an external magnetic field, nanoparticle concentration, chemical reaction, Hall current, and ion slip conditions. The governing equations along with the boundary conditions are modelled under the long wavelength assumption. The solutions are obtained analytically using the homotopy perturbation technique, and the physical interpretation of the pertinent parameters is discussed. Diagrammatic sketches are given for the physical expressions with the relevant parameters considered in the flow field. The primary findings can be outlined as follows:

(i) An increment in the Brownian parameter *N*_b_ causes an increase in the thermal conductivity *θ*, but causes a decrease in the concentration profile *Φ*.

(ii) For a relatively large value of *N*_b_, there is a dual behaviour of the chemical reaction *γ* on the concentration profile.

(iii) The effect of the thermophoresis *N*_t_ is to enhance *θ* and *Φ*.

(iv) The chemical reaction *γ* has a decreasing effect on both *θ* and *Φ*.

(v) Unlike the effect of the basic density Grashof number *G*_rT_, Hartmann number *M*, and Weissenberg number We, the slip parameter *β*_i_, Hall parameter *β*_e_, thermal Grashof number *G*_rF_, and power law index *n* serve to boost the velocity distribution markedly before a certain critical point.

(vi) The pressure increase Δ*p*_*L*_ is enhanced with an increase in *β*_i_, *β*_e_, We, and *G*_rT_, whereas it decreases with an increase in *G*_rF_, *M*, and *Q̄*.

(vii) In the absence of the magnetic field, the pressure increase attains the highest value, and the friction force attains the lowest value.

(viii) There is a critical point of *M* at which the behaviours of the pressure increase and friction force are reversed for the progressive values of *n*.

(ix) Contrary to the influence of *β*_i_, *β*_e_, We, and *G*_rT_ on the friction force Δ*f*_*L*_, *G*_rF_, M, and *Q̄* are seen to enhance Δ*f*_*L*_ prominently.

(x) Setting *n* = 0, We = 0, *M* = 0, and *G*_rT_ = *G*_rF_ = 0 in our analysis, the intrinsic equations governing the flow of Gupta^39^ are recovered.

(xi) Our study agrees with that reported for Newtonian fluid by Srivastava and Srivastava^36^ in the absence of the magnetic field, basic and thermal Grashof numbers, and Weissenberg number.

(xii) Upon solving our model in plane or axisymmetric geometries for *n* = 0, We = 0, *M* = 0, and *G*_rT_ = *G*_rF_ = 0 implies to a consistent physical situation as discussed by Shapiro and Jaffrin.^35^

(xiii) Upon adopting the analysis of Mekheimer^2^ for the Newtonian fluids, our fundamental equations will be in a perfect match with his after setting *n* = 0, We = 0, *M* = 0, and *G*_rT_ = *G*_rF_ = 0 in our analysis.

(xiv) Choosing *n* = 0, *M* = 0, and *γ* = 0 in the present investigation, our system of equations coincides with that of Abbas *et al.*^40^ for the Newtonian fluids.

(xv) If *n*, *β*_i_, and *β*_e_ vanish in the leading equations that govern the current flow field, the system is reduced to that of Rashidi *et al.*^41^ if performed in a non-porous medium in the absence of the radiation parameter, heat source/sink, and Weissenberg number.

## Conflicts of interest

There are no conflicts to declare.

## Nomenclature

*ũ*,*ṽ*: Velocity components (m s^−1^)
*x̃*,*ỹ*: Cartesian coordinate (m)
*p̃*
: Pressure in fixed frame (N m^−2^)
*ã*
: Wave amplitude (m)
*b*(*x̃*): Width of the channel (m)
*c̃*
: Wave velocity (m s^−1^)
*b*_0_
: Half width at the inlet
Pr: Prandtl number
Re: Reynolds number
*t̃*
: Time (s)
*G*_rF_
: Basic density Grashof number
*G*_rT_
: Thermal Grashof number
*N*_b_
: Brownian motion parameter
*N*_t_
: Thermophoresis parameter
*K̄*(≪1): Constant
*n*
: Power law index
*B*_0_
: Magnetic field (in Tesla)
We: Weissenberg number
*Q*
: Volume flow rate (m^3^ s^−1^)
*T*, *F*: Temperature (K) and concentration
*T*_0_, *T*_1_: Temperature at the center and at the wall
*F*_0_, *F*_1_: Nanoparticle fraction at the center and at the wall
*q̃*
: Perturbation parameter
*M*
: Hartmann number
*g*
: Acceleration due to gravity (m s^−2^)
*D*_B_
: Brownian diffusion coefficient (m^2^ s^−1^)
*D*_T_
: Thermophoretic diffusion coefficient (m^2^ s^−1^)
*E*
: Electric field (V m^−1^)
*V*
: Fluid velocity (m s^−1^)
*j*
: Current density (A m^2^).

### Greek characters

*γ*
: Chemical reaction parameter
*κ*
: Nanofluid thermal conductivity (W m K^−1^)
*β*
: Heat source/sink parameter
*μ*
: Viscosity of the fluid (N s m^−2^)
*θ*
: Dimensionless temperature profile
*Φ*
: Nanoparticle concentration
*σ*
: Electrical conductivity (S m^−1^)

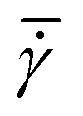

Second invariant tensor.
*δ*
: Wavenumber (m^−1^)
*c*_p_
: Effective heat capacity of nanoparticle (J/K)
*ν*
: Nanofluid kinematic viscosity(m^2^ s^−1^)
(*ρ*)_p_: Nanoparticle mass density (kg m^−3^)
*ρ*_f_
: Fluid density (kg m^−3^)
*ρ*_f_0__
: Fluid density at the reference temperature (*T*_0_) (kg m^−3^)
*ζ*
: Volumetric expansion coefficient of the fluid
(ρc)_f_: Heat capacity of fluid (J/K)
*λ*
: Wavelength (m)
*ϕ*
: Amplitude ratio
*η*_∞_
: Infinite shear rate viscosity
*η*_0_
: Zero shear rate viscosity
*Γ*
: Time constant
*ω*_e_
: Cyclotron frequency
*τ*_e_
: Electron collision time
*β*_i_
: Ion slip parameter
*β*_e_
: Hall parameter.

## Supplementary Material
